# Interaction of *Coxiella burnetii* Strains of Different Sources and Genotypes with Bovine and Human Monocyte-Derived Macrophages

**DOI:** 10.3389/fcimb.2017.00543

**Published:** 2018-01-12

**Authors:** Katharina Sobotta, Kirstin Hillarius, Pablo H. Jiménez, Katharina Kerner, Carsten Heydel, Christian Menge

**Affiliations:** ^1^Friedrich-Loeffler-Institut, Institute of Molecular Pathogenesis, Jena, Germany; ^2^Chemisches und Veterinäruntersuchungsamt Karlsruhe, Karlsruhe, Germany; ^3^Institute for Hygiene and Infectious Diseases of Animals, Justus-Liebig-University, Giessen, Germany

**Keywords:** *Coxiella burnetii*, genotype, *in vitro*, host cell response, MLVA

## Abstract

Most human Q fever infections originate from small ruminants. By contrast, highly prevalent shedding of *Coxiella* (*C*.) *burnetii* by bovine milk rarely results in human disease. We hypothesized that primary bovine and human monocyte-derived macrophages (MDM) represent a suitable *in vitro* model for the identification of strain-specific virulence properties at the cellular level. Twelve different *C. burnetii* strains were selected to represent different host species and multiple loci variable number of tandem repeat analysis (MLVA) genotypes. Infection efficiency and replication of *C. burnetii* were monitored by cell culture re-titration and qPCR. Expression of immunoregulatory factors after MDM infection was measured by qRT-PCR and flow cytometry. Invasion, replication and MDM response differed between *C. burnetii* strains but not between MDMs of the two hosts. *S*trains isolated from ruminants were less well internalized than isolates from humans and rodents. Internalization of MLVA group I strains was lower compared to other genogroups. Replication efficacy of *C. burnetii* in MDM ranged from low (MLVA group III) to high (MLVA group IV). Infected human and bovine MDM responded with a principal up-regulation of pro-inflammatory cytokines such as IL-1β, IL-12, and TNF-α. However, MLVA group IV strains induced a pronounced host response whereas infection with group I strains resulted in a milder response. *C. burnetii* infection marginally affected polarization of MDM. Only one *C. burnetii* strain of MLVA group IV caused a substantial up-regulation of activation markers (CD40, CD80) on the surface of bovine and human MDM. The study showed that replication of *C. burnetii* in MDM and the subsequent host cell response is genotype-specific rather than being determined by the host species pointing to a clear distinction in *C. burnetii* virulence between the genetic groups.

## Introduction

*Coxiella* (*C*.) *burnetii*, a gram-negative obligate intracellular bacterium, is the causative agent of Q fever, a widely distributed zooanthroponosis. After an incubation time of 2 weeks, Q fever can manifest as acute, self-limiting flu-like illness with complications like pneumonia and hepatitis. Chronic disease occurs more rarely and presents as, e.g., endocarditis or fatigue syndrome (Maurin and Raoult, 1999; Sobotta et al., 2016). Pregnant women are at high risk for development of chronic Q fever which can result in adverse pregnancy outcome with, e.g., spontaneous abortion or intrauterine fetal death (Carcopino et al., 2009). The most common sources for *C. burnetii* transmission to humans are domestic ruminants. Humans become infected by aerosols derived from contaminated abortion material, birth products, urine or feces. The threat ruminant *C. burnetii* carriers pose to humans was dramatically illustrated by a large Q fever outbreak in the Netherlands. During this outbreak more than 4,000 human cases were notified between 2007 and 2011 and approximately 52,000 ruminants were culled as part of the countermeasures taken to control the epidemic (van der Hoek et al., 2012). Three *C. burnetii* strains, which occurred both in humans and in ruminants, were isolated during this outbreak (Tilburg et al., 2012).

To avoid further spread of *C. burnetii* in herds or transmission to humans, presumptive differences in the virulence and host adaptation of different *C. burnetii* strains must be considered. *C. burnetii* strains exhibit a high genetic diversity and differ in their restriction fragment length polymorphism (RFLP) pattern (Hendrix et al., 1991; Beare, 2012). Jäger et al. showed that RFLP-groups correlated with the origin and the geographic distribution of *C. burnetii* (Jager et al., 1998). The high similarity of the genetic profile of members of RFLP group I which includes reference strain Nine Mile (NMI) was taken as evidence to suggest the presence of a globally extended common ancestor, which changed its genetic properties only slightly in the respective areas (Beare et al., 2006). Hendrix et al. arranged 32 *C. burnetii* strains into 6 RFLP groups (I–VI) (Hendrix et al., 1991) and unveiled a relationship between the RFLP group and the virulence of respective strains in a rodent model of acute Q fever. Strains within the same genogroup caused the same pathology in guinea pigs and similar cytokine patterns in response to *C. burnetii* infection in a mouse model (Russell-Lodrigue et al., 2009).

New typing methods may help to define correlations between the genotype and virulence of *C. burnetii* more precisely. MLVA (multiple loci variable number of tandem repeat analysis) is a molecular typing method to subtype bacterial isolates based on variable tandem repeats. Svraka et al. selected eight tandem repeat loci from the completely sequenced genome of the reference strain Nine Mile RSA 493 and were the first to apply the MLVA method for the classification of *C. burnetii* strains (Seshadri et al., 2003; Svraka et al., 2006). Jiménez used the method of Svraka to classify 103 *C. burnetii* strains into 4 genetic groups (I–IV), which correlated with the genetic properties, origin of the strains and indirectly with disease pattern (Jiménez, 2012). Members of genogroup I and IV provoked acute *C. burnetii* infections, whereas chronic Q fever infections were often associated with MLVA group II strains (Jiménez, 2012). *Coxiella burnetii* strains normally possess one of four autonomously replicating plasmids termed QpH1, QpRs, QpDV, and QpDG, or a chromosomally integrated QpH1-like plasmid. QpH1 plasmids are closely related and likely identical to QpDG (Mallavia, 1991; Valkova and Kazar, 1995; Willems et al., 1997; Jager et al., 2002). MLVA groups I, III, and IV were found to be associated with a QpH1 plasmid while MLVA group II strains were very heterogeneous in their plasmid content. Within the collection of 103 strains, members of MLVA group I, III and IV possessed the *adaA* gene which encodes for the polypeptide acute disease antigen A described to be primarily present in strains isolated from acutely diseased patients (Zhang et al., 2005). Strains which cause a chronic Q fever infection do not harbor the *adaA* gene (Frangoulidis et al., 2009). However some strains without *adaA* but epidemiologically linked to acute infection could also be identified questioning the usefulness of *adaA* as biomarker for *C. burnetii* virulence (Jiménez, 2012).

Macrophages are generally regarded the most important host cells for *C. burnetii* (Maurin and Raoult, 1999). In bovine and human macrophages, *C. burnetii* replicates in parasitophorous vacuoles (PV) with lysosomal acidic characteristics (Sobotta et al., 2016). Infection of these cells induces an early pro-inflammatory immune response by increased expression of cytokines such as IL-12, TNF-α, and IL-1β (Tujulin et al., 1999; Graham et al., 2013; Sobotta et al., 2016). We recently suggested that the early stimulation (3 h *p. i*.) of bovine monocyte derived macrophages (MDM) during *C. burnetii* infection mainly depends on surface binding of bacteria or bacterial compounds to host cell receptors rather than bacterial replication. We hypothesized that the MDM culture model is suitable for characterizing the bacterial replication and host cell response after infection with *C. burnetii* (Sobotta et al., 2016). The MDM model may be used for the identification of strain-specific virulence properties avoiding the application of the elaborate and less ethical rodent model of acute Q fever (Russell-Lodrigue et al., 2009). We therefore inoculated MDM of bovine and human origin with 12 heterogeneous *C. burnetii* strains from MLVA groups I to IV selected from a well characterized strain collection and assessed *C. burnetii* invasion and replication as well as resulting host cell responses.

## Materials and methods

### Preparation and characterization of bacterial inocula

Bacterial strains deployed in this study are listed in Table 1. All strains were selected from the strain collection of the Institute for Hygiene and Infectious Diseases of Animals (Giessen) except 5 strains (Cb 23/2, Cb 71/3, Cb 98/2, Cb 19/34, Cb 30/14) which were provided by the German National Reference Laboratory for Q fever (Jena, Germany). Strain Z69/06 (strain 6) is a mixed culture of 2 *C. burnetii* strains with different genotypes (III and IV). The preparation of the inocula and the characterization of the strains were already described (Jiménez, 2012; Sobotta et al., 2016). MLVA genotyping was conducted using flanked primers for seven previously described microsatellites (Svraka et al., 2006; Jiménez, 2012). For each PCR reaction, 1 μl of the respective primer solution (Table 2, f.c. 0.2 μM/primer), 1 μl template (1 × 10^8^ *C. burnetii* cells in 0.9% NaCl solution) and 3 μl deionized water were added to 5 μl 2x QIAGEN Multiplex PCR Master Mix (Qiagen, Hilden, Germany). PCR was performed in a T1 Thermocycler (Biometra, Germany). Incubation for 15 min at 95°C was followed by 25 cycles (Cox4-PCR 35 cycles) comprising incubation for 30 s at 95°C, 90 s at primer pair-specific temperature (Jiménez, 2012), and 30 s at 72°C. Amplification was completed by incubation for 10 min at 72°C. PCR products were separated in 6% polyacrylamide gels on a LI-COR 4200 DNA Sequencing System. The smaller amplificates of the Cox4-PCR were separated in 5% polyacrylamide gels in a Minigel-Twin-Electrophoresis chamber (Biometra, Göttingen, Germany). Sizes of bands were analyzed using GenImagIR (Scanalytics).

**Table 1:** *Coxiella burnetii* strains used in this study.

***C. burnetii* strain**	**Strain no**.	**Genetic properties**			**LPS phase^a^**	**Infection of host^b^**		**Immediate host**
		**MLVA group**	**Plasmid**	***adaA***		**Disease**	**Course**	
Nine Mile I (493)^c^	1	IV	QpH1	+	I ≈ II	u.	u.	Tick
Henzerling	2	IV	QpH1	+	I	Pneumonia	Acute	Human
Scurry	3	II	No plasmid	–	I > II	Hepatitis	Chronic	Human
Dugway	4	III	QpH1^d^	+	I ≈ II	u.	u.	Rodent
Z3055/91	5	II	QpH1	+	I < II	u.	u.	Sheep
Z69/06	6	III	QpH1	+	I < II	u.	u.	Cattle
Cb 23/2	7	I	QpH1	+	I < II	u.	u.	Sheep
Cb 71/3	8	III	QpH1	+	I < II	u.	u.	Goat
Cb 98/2	9	III	QpH1	+	I ≈ II	u.	u.	Cattle
Cb 19/34	10	I	QpH1	+	I < II	u.	u.	Goat
Cb 30/14	11	I	QpH1	+	I > II	u.	u.	Sheep
Z3464/92	12	I	QpH1	+	I ≈ II	abortion	u.	Goat

a *I ≈ II = comparable amounts of Phase I and II LPS as deduced from band intensities in silver-stained SDS-PAGE gels; I > II = higher amounts of Phase I LPS; I < II = higher amounts of Phase II LPS*.

b *u. = unknown*.

c *Nine Mile I is included only as reference strain. All data of Nine Mile I in this manuscript have been published previously (Sobotta et al., 2016)*.

d *Dugway strain 5J108-11 reportedly contains a QpDG plasmid. However, of the 3 strains of Dugway available we used a strain which did not carry the QpDG plasmid*.

**Table 2:** Sequences of MLVA primers.

**Primer**	**Sequence 5′-3′**
Cox1	F^*^: AGA AAA AAG CAC AGA CCT TGA R: TTC CTG ATT TAA AAG GGT GAC T
Cox2	F^*^: TTC TTT ATT TCA GGC CGG AGT R: CCG GTA ACG CCG ATT AGT AA
Cox3	F^*^: GCA ATC CAG TTG GAA AGA A R: ATT GAA GTA ATC CAT CGA TGA TT
Cox4	F^*^: ATG AAG AAA GGA TGG AGG G R: TGC AAG GAT AGC CT
Cox5	F^*^: AAT GGA GTT TGT TAG CAA AGA AA R: AAA GAC AAG CAA AAC GAT AAA AA
Cox6	F^*^: GAC AAA AAT CAA TAG CCC GT R: GAG TTG TGT GGC TTC GC
Cox7	F^*^: ACA GGC CGG TAT TCT AAC C R: CCT CAG CAC CCA TTC AG

* *DY-781*.

The plasmid status of *C. burnetii* strains was determined by mulitplex PCR (Jiménez, 2012). Reactions contained 5 μl of 2x Multiplex PCR Master Mix (Qiagen, Germany), 1 μl of gel loading dye (25 g sucrose, 0.3 g Orange G, deionized water ad 100 ml), 2 μl of deionized water, 1 μl primer mix (Table 3, f.c. 0.2 μM each) and 1 μl of template-DNA (1 × 10^8^ *C. burnetii* cells). The PCR reaction profile was: a start denaturation step (15 min at 95°C), 35 cycles (95°C for 30 s, 58°C for 90 s, and 72°C for 30 s) and a final extension step at 72°C for 10 min. Products were separated in 2% agarose gels and visualized via Midori Green. Detection of the *adaA* gene was performed as described by Zhang et al. (2005).

**Table 3:** Sequences of plasmid primers.

**Primer**	**Sequence 5′-3′**
QpDV	F: TTC TTA GTA ACC GGT AGT GGA TGT CC R: GGC TGT TGT GCA TAT TAG TGT GAT G
QpH1	F: GCG AGT TGA GGC AGA AGA GG R: GCA CGG TAG AAT GGA AGG AAG
QpRS	F: CTT TCT AAT GGG ATT CCG TCA GC R: AGT ATT CAA TTA AGG ACA CCC GTC A
chromosomally integrated QpH1-like plasmid	F: GCA TGC TCC ATA GCC AAC GTA ATC T R: TGC AAT TCT GTT GTT ATC AGT GCC T

As most *C. burnetii* strains where passaged several times on Buffalo Green Monkey cells, lipopolysaccharide (LPS) composition of each inoculum after each passage was analyzed for changes in the LPS profile. After extraction (Kersh et al., 2011), relative abundances of full-length and truncated LPS molecules forming the cell envelope of the *C. burnetii* strains were determined by sodium dodecyl sulfate-polyacrylamide gel electrophoresis (4–20% Tris/Glycine gel) and subsequent silver staining of the gels. Ratio of full-length and truncated LPS was estimated by image tonality (light intensity of LPS bands).

### Establishment of monocyte-derived macrophages (MDM) from bovines and humans

Bovine MDM (boMDM) were isolated from the blood of healthy Holstein Friesian cattle following a protocol described before (Sobotta et al., 2016). For preparation of human MDM (huMDM), peripheral blood mononuclear cells (PBMC) were isolated from healthy blood donor's using density gradient centrifugation. Briefly, buffy coats were obtained from the Institute for Transfusion Medicine, University Hospital of Jena. RPMI 1640 medium was supplied by PAA/PAN-Biotech (Aidenbach, Germany) and pools of human sera were supplied by Sigma-Aldrich (St. Lewis, USA). Human PBMC were isolated from buffy coats according to the technique adapted from Pinet et al. (2003). In brief, 40 ml of buffy coat diluted with PBS (1:1, vol/vol) were carefully loaded onto a Pancoll human gradient (PAA/PAN-Biotech). After an initial centrifugation step (45 min, 250 × *g*), the interphase was collected and washed firstly twice with PBS containing 0.1% EDTA (100 × *g*, 340 × *g* for 25 min) and then 2 times with plain PBS (290 × *g*, 160 × *g* for 25 min). Finally, the cell pellet was resuspended in an RPMI 1640 culture medium containing penicillin (10,000 U/ml), streptomycin (10,000 μg/μl), FBS (5%, Gibco®-ThermoFisher Scientific, Waltham, USA) and human serum (5%, Si070, Sigma-Aldrich). The cells were distributed at a density of 1 × 10^7^ and 1 × 10^6^ cells per well in 6-well and 24-well culture plates (Advanced well dishes, Greiner bio-one, Kremsmünster, Austria), respectively. After 90 min of adherence, the non-adherent cells were seeded in a fresh well. The adherent cells were washed twice with PBS and RPMI 1640 culture medium was added. Culture medium was changed every 2 days. At day 6–8, the cells were counted and used for further experiments. For each preparation, the cell composition of MDM cultures was determined by FACS analysis (see below). BoMDM and huMDM cultures consisted of 81 and 85% CD14^+^ cells, respectively, with light scatter characteristics (size and granulation) of macrophages.

All animal experiments were conducted according to the rules laid down in the German Animal Protection Act and approved by the competent authority (Thuringian State Office for Consumer Protection, reg. no. 22-2684-04-04-102/13 and reg. no. 04-004/1). The use of patient's blood was endorsed by the ethics committee of the university hospital Jena (reg. no. 3058-02/11).

### Cultivation of MDM for studying *C. burnetii* invasion and replication kinetics

All infection experiments were conducted under biosafety level 3 conditions. Invasion and replication of *C. burnetii* strains were studied with boMDM (5 × 10^5^ cells/well) cultivated in polysterol tubes (Greiner) and huMDM (approximately 5 × 10^5^ cells/well) cultivated in 24-well culture plates (Advanced, Greiner). MDM cultures were inoculated at a multiplicity of infection (Voth et al., 2011) of 100 by addition of bacteria to IMDM culture medium (IMDM without phenol red, 2% FCS, 0.05% 100 mM β-mercaptoethanol) or RPMI 1640 culture medium without antibiotics for 1 h (37°C, 5% CO_2_). After inoculation, MDM were washed 3 times with 0.89% NaCl solution and replenished with medium. Triplicate cell cultures were harvested by three freeze/thaw cycles at 1, 7, and 14 d *p. i* to monitor *C. burnetii* invasion and replication efficacies, respectively. Invasion and replication was quantified by determination of median tissue culture infective doses (TCID_50_) in the cellular fraction of the cultures by endpoint titration on BGM cells or by isocitrate dehydrogenase (*icd*) PCR as described elsewhere (Sobotta et al., 2016). The replication efficiency was calculated by dividing the TCID_50_ values obtained 14 d *p. i*. by the respective values obtained 1 d *p. i*.

### Cultivation of MDM for RNA isolation

MDM (2 × 10^6^ cells/well) were cultured in 6 well culture plates (Greiner) and inoculated with *C. burnetii* strains in biological triplicates as described above. As controls, cells were mock infected with 0.89% NaCl solution (as negative control) or stimulated with LPS of *E. coli* O111:B4 (6 μg/ml) as positive control. Cells were lysed with RLT buffer (RNeasy Mini Kit [Qiagen, Hilden, Germany]) at 3 h *p. i*. and total RNA was isolated with the RNeasy Mini Kit (Qiagen) according to the instructions of the manufacturer. To avoid DNA contamination, RNA was purified with the RNase-free DNase set (Qiagen).

### Reverse transcription and cytokine-specific real-time PCR

Equal RNA amounts from each sample were reversely transcribed into cDNA as described elsewhere (Sobotta et al., 2016). Levels of gene expression of different host-specific cytokines and GAPDH as a house keeping gene were determined in technical duplicates by quantitative real-time SYBR Green-based (Applied Biosystem, Waltham, USA) PCR using ABI Prism®7500 (Applied Biosystem). All primers (Table 4) were exon-intron spanning and run at an annealing temperature of 60°C. PCR reaction profile was: denaturation (10 min, 95°C), annealing (1 min, 60°C; 39 cycles) and melting step (15°C, 60°C). PCR products yielding detectable signals later than cycle 38 were graded non-detectable. Relative gene expression levels were calculated by using relative expression software REST (Pfaffl et al., 2002).

**Table 4:** Sequences of cytokine and chemokine primers.

**Primer**	**Specificity**	**Sequence 5′–3′**
GAPDH	Bovine	F: GCG ATA CTC ACT CTT CTA CCT TCG A R: TCG TAC CAG GAA ATG AGC TTG AC
	Human	F: TGG GTG TGA ACC ATG AGA AG R: GCT AAG CAG TTG GTG GTG C
IL-1β	Bovine	F: ACC TGA ACC CAT CAA CGA AAT G R: TAG GGT CAT CAG CCT CAA ATA ACA
	Human	F: TGA TGG CTT ATT ACA GTG GCA ATG R: GTA GTG GTG GTG GGA GAT TCG
IL-8	Bovine	F: CAC TGT GAA AAA TTC AGA AAT CAT TGT TA R: CTT CAC CAA ATA CCT GCA CAA CCT TC
	Human	F: TCC TGA TTT CTG CAG CTC TGT R: AAT TTC TGT GTT GGC GCA GT
IL-10	Bovine	F: GTG ATG CCA CAG GCT GAG AA R: TGC TCT TGT TTT CGC AGG GCA
	Human	F: GGT TGC CAA GCC TTA TCG GA R: ACC TGC TCC ACT GCC TTG CT
IL-12	Bovine/Human	F: GCA GCT TCT TCA TCA GGG ACA T R: CCT CCA CCT GCC GAG AAT T
INF-γ	Bovine	F: TTC TTG AAC GGC AGC TCT GAG R: TGG CGA CAG GTC ATT CAT CA
	Human	F: CCA ACG CAA AGC AAT ACA TGA R: CCT TTT TCG CTT CCC TGT TTT
MCP1	Bovine	F: GCT GTG ATT TTC AAG ACC ATC CT R: GGC GTC CTG GAC CCA TTT
	Human	F: GTG CAG AGG CTC GCG AGC TA R: CAG GTG GTC CAT GGA ATC CTG
RANTES	Bovine	F: CAT GGC AGC AGT TGT CTT TAT CA R: CTC TCG CAC CCA CTT CTT CTC T
	Human	F: AAC CCA GCA GTC GTC TTT GTC A R: CTC CCG AAC CCA TTT CTT CTC T
TGF-β	Bovine	F: GGC CCT GCC CTT ACA TCT G R: CGG GTT GTG CTG GTT GTA CA
	Human	F: GGC CCT GCC CCT ACA TTT G R: CGG GTT ATG CTG GTT GTA CA
TNF-α	Bovine	F: TCT TCT CAA GCC TCA AGT AAC AAG T R: CCA TGA GGG CAT TGG CAT AC
	Human	F: TCT TCT CGA ACC CCG AGT GA R: CCT CTG ATG GCA CCA CCA G

### Cultivation of MDM for flow cytometry analysis

MDM (2 × 10^6^ cells/well, 6 well plates) were inoculated with *C. burnetii* (MOI 100 for 1 h) or stimulated with LPS of *E. coli* O111:B4 (6 μg/ml). After incubation for 24 h, cells were detached by incubation with Accutase® (PAA/PAN-Biotech), transferred into microtiter plates (V-shape; Greiner) and pelletized by centrifugation (400 × *g*, 4 min and 4°C). For detection of surface proteins (CD40, CD80, CD86, MHCI, and II) cells were incubated with 50 μl diluted primary antibody or directly labeled antibodies for 20 min (Table 5). After washing (washing buffer: PBS, 0.5% FKS), cells were fixed with paraformaldehyde (4% in PBS, 4°C, 24 h) for decontamination and transfer into BSL2 laboratory. Thereafter, cells were washed and incubated with 1:1,000 secondary antibody (anti-mouse IgG1-APC, anti-mouse IgG2a-APC, anti-mouse IgG2b-PE; Southern Biotech, USA) diluted in PBS for 20 min. Finally cells were washed again and analyzed with BD FACSCanto™ II (Becton Dickinson [BD], Heidelberg, Germany). Data were evaluated with BD FACSDIVA™ software (version 6).

**Table 5:** Antibodies against MDM surface antigens used in this study.

**Antigen**	**Species**	**Antibody clone (source)**	**Isotype**	**Working dilution**
CD14	Bovine	CAM36A (VMRD, Pullman, WA, USA)	IgG1	1:500
CD14 (FITC)	Human	61D3 (Biozol)	IgG1	1:222
CD40	Bovine	IL-A156 (Dirk Werling^a^)	IgG1	1:10
CD40 (FITC)	Human	5C3 (BD Biosciences)	IgG1κ	1:222
CD80	Bovine	IL-A159 (Dirk Werling)	IgG1	1:10
CD80 (FITC)	Human	MEM-233 (Biozol, Eching, Germany)	IgG1	1:222
CD86	Bovine	IL-A190 (Dirk Werling^a^)	IgG1	1:10
CD86 (FITC)	Human	BU63 (Biozol)	IgG1	1:222
MHCI	Bovine	PT85A (VMRD, Pullman, WA, USA)	IgG2a	1:500
MHCI (FITC)	Human	G46-2.6 (BD Biosciences)	IgG1κ	1:400
MHCII	Bovine	H34A (VMRD, Pullman, WA, USA)	IgG2b	1:500
MHCII (R-PE)	Human	423L (Biozol)	IgG2a	1:400

a *Royal Veterinary College, Hatfield, UK*.

### Statistical analyses

Statistical comparisons were conducted using *t*-test or Mann-Whitney *U*-test with statistic software XLSTAT. *P*-value of ≤ 0.05 (“^*^”) show a statistically significant difference at the 95% confidence level, a *P*-value of ≤ 0.01 (“^**^”) at the 99% confidence level. Real-time PCR data was analyzed by a randomization test with pairwise reallocation (software REST, Pfaffl et al., 2002). For statistical comparisons between MLVA genotypes, origin or LPS-type all single results of each strain were grouped and analyzed using appropriate statistical test.

## Results

### *Coxiella burnetii* strains were internalized by boMDM and huMDM with different efficiencies

**Figure 1:**
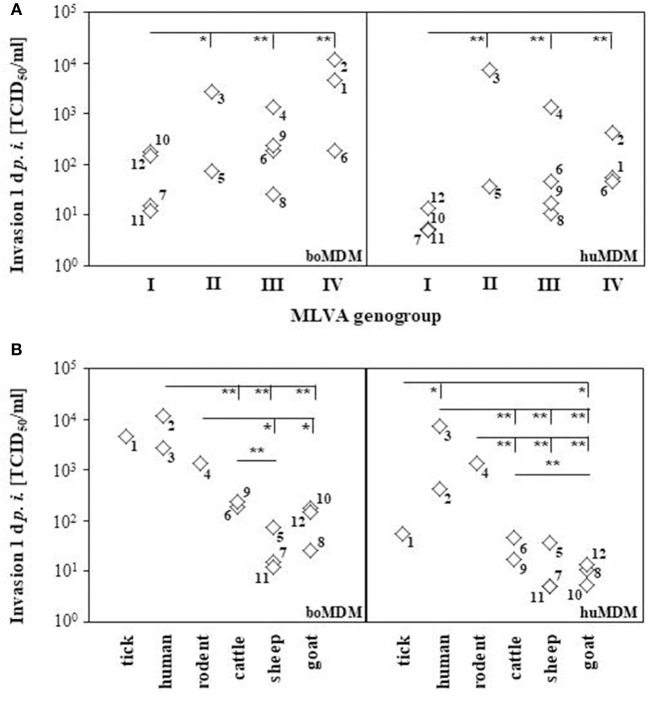
Internalization of *C. burnetii* strains in bovine and human MDM. Cells were inoculated with *C. burnetii* strains (100 MOI, 1 h). The number of cell-associated *C. burnetii* bacteria was quantified by TCID_50_ assay at 1 d *p. i.*. Mean values derived from three independent experiments per strain were grouped according to the MLVA genotype **(A)** or the immediate host the strains were isolated from **(B)** and depicted as diamond blot (numbers next to the diamonds refer to the number of the strain as listed in Table 1). Statistical analyses were calculated using all single values of each experiment (^*^p ≤ 0.05; ^**^p ≤ 0.01; left hand ends of horizontal lines indicate the data sets used for respective comparison).

In human and bovine MDM cultures, numbers of cell-bound bacteria were determined for each strain 1 d *p. i*. by *icd* PCR and titration on BGM cells. All *C. burnetii* strains were internalized into bovine and human MDM. Determination of total bacterial numbers in terms of genome equivalents by PCR yielded no significant differences in the number of invaded bacteria between the *C. burnetii* strains in both MDM cultures (data not shown). By contrast, the number of viable *C. burnetii* bacteria re-titrated from infected MDM cultures markedly differed between the strains (Figure 1). To identify parameters indicative of invasion differences of *C. burnetii* strains in bovine and humane MDMs, we compared the LPS-types, the origin of the strains and their genotypes with the viable cell-bound bacteria measured by TCID_50_ assay. Since most of the strains included in this study contain LPS molecules of both chemotypes, phase I and II, in different relative quantities (data not shown), no clear conclusion can be drawn on the influence of LPS on the invasion. Moreover, the origin of strains and the genotype correlated with the ability to successfully enter host cells. *Coxiella burnetii* strains belonging to MLVA groups II to IV invaded human and bovine MDM significantly more efficiently than MLVA group I strains which were poorly internalized by boMDM and even less by huMDM (Figure 1A). Different from relative replication efficiencies (see below), invasiveness of group III and group IV strains did not markedly differ. The most prominent difference was found when the strains were grouped according to their initial host (Figure 1B). Isolates from ruminants were significantly less well internalized by human and bovine MDM than human and rodent isolates.

### *Coxiella burnetii* strains vary in their replication efficacy in boMDM and huMDM

**Figure 2:**
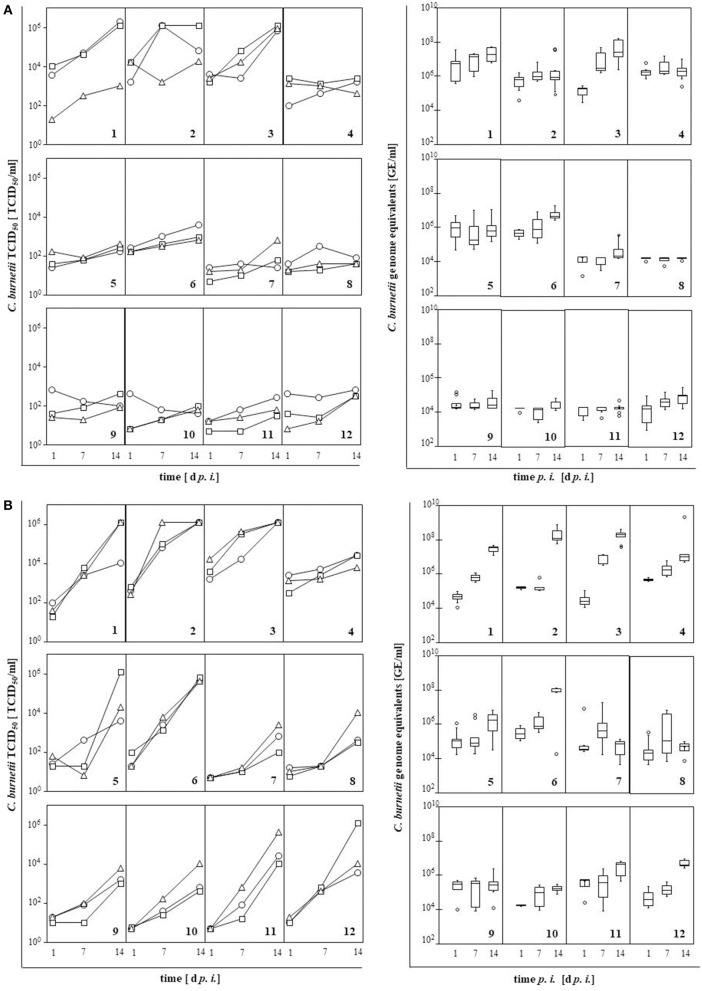
Replication of *C. burnetii* strains in bovine **(A)** and human MDM **(B)** measured by TCID_50_ assay (left) and qPCR (right). Cells were inoculated with *C. burnetii* strains (100 MOI, 1 h). Numbers of viable bacteria (TCID_50_ assay, *n* = 3) and of genome equivalents (GE; qPCR, *n* = 6) were quantified at 1, 7, and 14 day *p. i.*. Results per *C. burnetii* strain are shown as individual curves or as box-whisker plots. Strains are indicated by numbers as listed in Table 1.

In MDM cultures of both host species, numbers of viable bacteria were monitored for each strain over a period of 14 d by TCID_50_ assay and qPCR (Figure 2). Replication efficiency varied with strain and host. Generally, *C. burnetii* strains replicated faster in huMDM than in boMDM. Bacterial numbers increased by 1–4 orders of magnitude in huMDM but only 0.5–2.6 orders of magnitude in boMDM. The numbers of genome equivalents (GE) determined by qPCR was two or more orders of magnitude higher than the numbers of viable bacteria measured by TCID_50_ but results obtained by qPCR revealed similar kinetics. While only strains 1 (“Nine Mile I”) and 3 (“Scurry”) showed a clear increase in the number of viable bacteria in boMDM, all other strains retained their viability in boMDM for up to 14 days but with little indication of replication neither by means of viable cell counts nor by genome counts. Except strain 4 (“Dugway”), all *C. burnetii* strains multiplied in huMDM leading to an increase in viable bacterial numbers by at least one order of magnitude. Different from the other strains, GE numbers did not increase or by less than one order of magnitude during infection of huMDM with strains 7 through 11.

**Figure 3:**
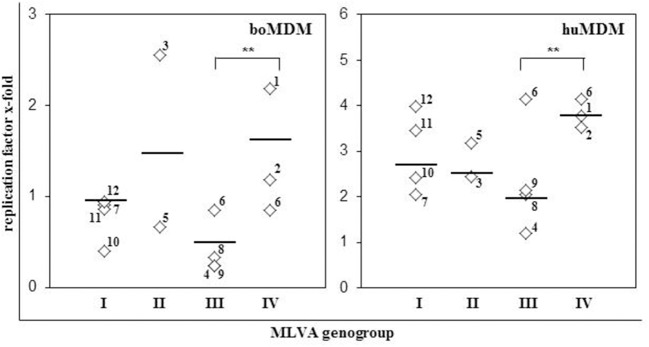
Genotype and replication efficacy of *C. burnetii* strains in bovine and human MDM. Increase of bacterial TCID_50_ was calculated as replication factor, i.e., fold increase in numbers of cell-associated *C. burnetii* bacteria between 1 and 14 day *p. i.*. Mean values of three different experiments per *C. burnetii* strain and median per MLVA group are presented (^**^p ≤ 0.01). Strains are indicated by numbers as listed in Table 1.

To identify parameters indicative of enhanced replication efficacy of *C. burnetii* strains, we compared the origin of strains and their genotype with the replication efficiency as assessed by quantitation of viable bacteria (TCID_50_). In MDM from both hosts, origin of *C. burnetii* isolates barely correlated with the slope of the replication curve (Figure 2, Table 1). Interestingly, replication efficiencies of *C. burnetii* strains in bovine and human MDM cultures differed between MLVA genotypes as well (Figure 3). Independent of the host species, strains of MLVA group IV replicated significantly better on average than strains belonging to group III with the latter isolates possessing the lowest efficacy of all genotypes assessed. Replication efficacies of group I and II strains ranged from moderate to high particularly in huMDM.

### *Coxiella burnetii* strains induced different immune responses in boMDM and huMDM

**Figure 4:**
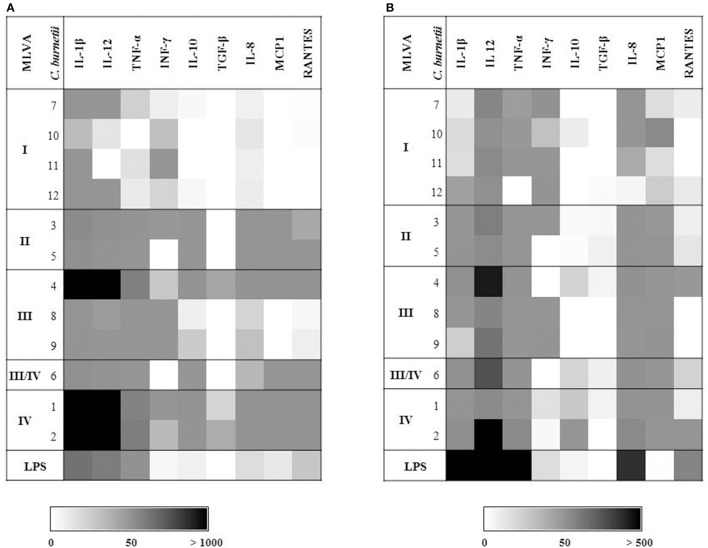
Influence of different *C. burnetii* strains on transcription of cytokines and chemokines by bovine **(A)** and human MDM **(B)**. Cells were inoculated with *C. burnetii* strains (100 MOI, 1 h) or stimulated with *E. coli* LPS (6 μg/ml) in biological triplicates. Amounts of cytokine- and chemokine-specific mRNA were determined 3 h *p. i*. in technical duplicates. Resulting mean values were normalized to GAPDH, calculated as fold change to cell control and expressed as averages of the values obtained from biological triplicates. Data is depicted in shades of gray as defined in the scale bar. Strains are indicated by numbers as listed in Table 1.

**Figure 5:**
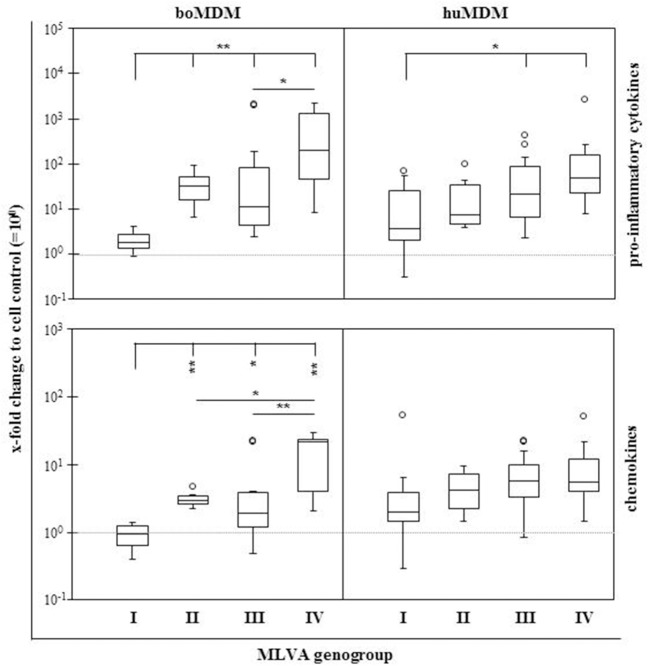
Effects of *C. burnetii* strains of different MLVA genogroups on transcription of cytokines and chemokines by bovine and human MDM. Cells were inoculated with *C. burnetii* strains (100 MOI, 1 h) and relative amounts of cytokine and chemokine-specific mRNA were determined 3 h *p. i*. as described in the legend to Figure 4. Resulting average values per target gene and *C. burnetii* strain were used to create combined data sets for proinflammatory cytokines (IL-1β, IL-12, and TNF-α) and for chemokines (IL-8, MCP1, and RANTES), respectively, and presented as box and whisker plots for each MLVA genogroup (^*^p ≤ 0.05; ^**^p ≤ 0.01). Dashed horizontal line indicates a fold increase of 1, i.e., equal to values from uninfected control cultures. Circles indicate values outside the 10th and 90th percentile.

*Coxiella burnetii* induces an early expression of pro-inflammatory cytokines in boMDM (Sobotta et al., 2016). As *C. burnetii* strains exhibit different invasion and replication efficiency when interacting with these cells, we additionally characterized the expression of immune mediators during the first 3 h *p. i*. (Figures 4A,B). Amounts of detected mRNA molecules specific for selected cytokines and chemokines differed between the strains and the source of the cells. While huMDM reacted stronger than boMDM to *E. coli* LPS, mRNA amounts of pro-inflammatory cytokines such as IL-1β (e.g., strain 1: 2,181-fold), IL-12 (e.g., strain 4: 2,117-fold) and TNF-α (e.g., strain H: 199-fold) particularly increased after *C. burnetii* infection of boMDM (Figure 4A). In contrast, anti-inflammatory cytokines, e.g., IL-10 and TGF-β were only slightly expressed. IL-10 was induced more strongly by some *C. burnetii* strains in boMDM (strain 1–6) than in huMDM (strain 2). TGF-β expression was only slightly elevated and following infection by strain 2 (2.2-fold) and 4 (2.3-fold) in boMDM, only. MDM of both species primarily differed in their *C. burnetii* strain-specific IL-10, IL-8, MCP1, and RANTES responses. Comparison between the strains within a species show marked differences in the expression of cytokines and chemokines between the genogroups. Comparisons of MLVA genotypes using combined data sets consisting of gene expression ratios of either pro-inflammatory cytokines (IL-1β, IL-12, TNF-α; Figure 5; upper graphs) or chemokines (IL-8, MCP1, RANTES; Figure 5; lower graphs) in the current investigation revealed a genogroup-specific expression which was fairly independent of the host species of the MDM. In boMDM in particular, induction of gene expression was most prominent by isolates of MLVA group IV and only marginally above levels of control cultures with members of group I. The same trend was observed for huMDM.

### *Coxiella burnetii* infection only marginally affected polarization of boMDM and huMDM

**Figure 6:**
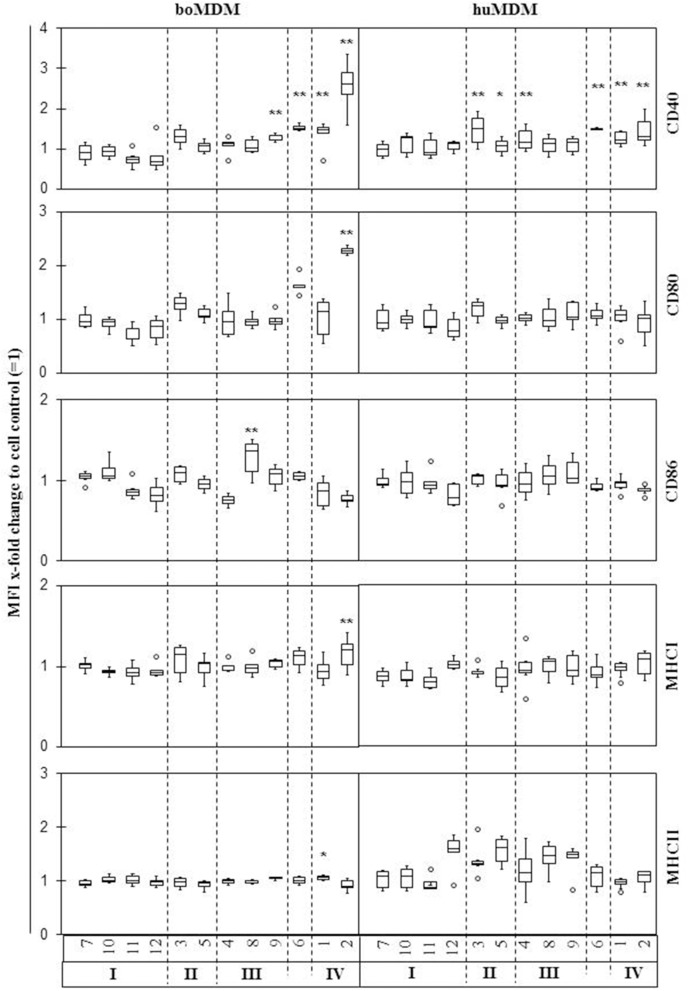
Effects of different *C. burnetii* strains on expression of different surface markers by bovine and human MDM. Cells were inoculated with *C. burnetii* strains (100 MOI for 1 h) or stimulated with *E. coli* LPS (6 μg/ml), harvested 24 h later and immunolabelled for surface markers CD40, CD80, CD86, MHCI, and MHCII. Results of 3–8 independent experiments per *C. burnetii* strain are shown as box and whisker plots (^*^p ≤ 0.05; ^**^p ≤ 0.01). Strains are indicated by numbers as listed in Table 1.

Macrophages respond to danger signals with a phenotypic change, e.g., expression of activation markers, to either display an M1 (e.g., CD40^+^, CD64^+^, CD80^+^, CD86^+^) or an M2 (e.g., CD163^+^, MHCII^high^) phenotype (Mosser and Edwards, 2008). To study whether infection with *C. burnetii* alters the differentiation process, expression of surface markers on human and bovine MDM was investigated 1 d *p. i*. (Figure 6). Incubation with *E. coli* LPS led to a clear increase in CD40 and CD80 expression in boMDM, and in CD40 expression of huMDM arguing in favor of a general responsiveness of the MDM cultures to microbe-associated molecular pattern. Nevertheless, phenotypic changes of MDM subsequent to *C. burnetii* infection were hardly detected in MDM cultures from both hosts. Only strain 2 (“Henzerling”) induced a significant increase of both, CD40 and CD80 protein expression, on the surface of boMDM. Despite some variations in MDM antigen expression levels after infection, a certain MDM reaction pattern could not be correlated with a particular *C. burnetii* genotype.

## Discussion

Genetic differences between *C. burnetii* strains affect their virulence and other biological properties to induce acute or chronic courses of infection (Beare et al., 2006). Results from studies in a rodent infection model imply that *C. burnetii* strains with same genotype cause similar pathological responses (Russell-Lodrigue et al., 2009). However, this model may only partially reproduce processes occurring in other hosts for *C. burnetii*. An infection model to study the host pathogen interaction of *C. burnetii* with ruminants *in vitro* was recently established by our group deploying boMDM cultures (Sobotta et al., 2016). Here, we used the model in a comparative approach with huMDM to assess the impact of different strain properties of *C. burnetii* on *in vitro* surrogates of virulence and host adaptation. To this end, 12 *C. burnetii* strains differing in genotype, origin and severity of the clinical course of Q fever were selected from a comprehensive strain collection (Jiménez, 2012).

Virulence is a quantitative property of bacterial strains describing their relative ability to cause disease in a given host species. *Coxiella burnetii* virulence is probably determined by several factors such as invasiveness, reproductive capability and the strength and character of the induced immune response. A comparison of the invasion rates of *C. burnetii* strains considered in this study unveiled a remarkable correlation with the origin of the isolate, i.e., the last host infected by the strains prior to isolation in the laboratory. Especially isolates from hosts not primarily considered sources of human infection such as human, rodents and ticks invaded more efficiently than isolates from the major recognized sources, i.e., sheep, goat, and cattle. Furthermore, the MLVA genotype seemed to be correlated with the invasiveness of strains. Isolates of genogroup I invaded clearly less than those of other genogroups. As MLVA is a measure to assess the phylogenetic relatedness of bacterial strains but does not inform on the possession of virulence-associated genes and their regulation, the molecular basis of these findings remain to be determined. Invasion of *C. burnetii* in phagocytic cells is regulated by receptors CR3 and α_V_β_3_ integrin. Both receptors are expressed on bovine (Sobotta et al., 2016) and human macrophages (Capo et al., 1999). In contrast, *C. burnetii* invasion in non-phagocytic cells is mediated by *Outer membrane protein A* (OmpA) (Martinez et al., 2014), a highly conserved outer membrane protein among gram-negative bacteria that is instrumental for adherence, invasion and activation of the host response (Confer and Ayalew, 2013). For *Enterobacteriales* the effect of OmpA is dependent on the concentration of this protein (Soulas et al., 2000). Consequently, it would be possible that differences in bacterial uptake rates by bovine and human MDM are a result of divergent expression of surface located invasion proteins or of secreted invasion proteins by the Coxiella type IV secretion system. Specific molecular biological characterization of *C. burnetii* isolates deploying the *in vitro* system used herein would offer the opportunity to identify virulence-associated genes which are instrumental for high level of adaptation to reservoir hosts and/or increased virulence for dead-end hosts like humans.

In this study, replication was monitored by counting viable bacteria (TCID_50_ assay) and genome equivalents (qPCR). The qPCR detected two orders of magnitude higher absolute numbers of bacterial genomes than the numbers of viable bacteria that were detected by TCID_50_ assay. A plausible explanation for this discrepancy is the fact that qPCR measures dead and VBNC (viable but not culturable) bacteria which apparently are abundant inside MDM cultures, i.e. cultures of cells which are professional phagocytes equipped to kill and digest bacterial pathogens. Interestingly, relative replication rates of the strains only slightly differed between both methods. Still, we consider the determination of viability and the infectivity by the TCID_50_ method more relevant to foster our understanding of infection processes *in vivo* as these also always mirror the balance between antimicrobial activity by the host and efficacy of invasion and replication realized by the pathogen.

*Coxiella burnetii* strains significantly differed in the replication rates. Generally *C. burnetii* replicated more efficiently in huMDM than in boMDM. On average, the replication of strains varied with the genotype and was less dependent on the strain origin or LPS-type. Strains of MLVA group IV showed a strong increase in bacterial numbers during cultivation for 14 days. Strains of group I and II replicated with a moderate to high and strains of group III with a very low efficiency. It was shown in infected guinea pigs that genotypes of *C. burnetii* strains imprint on the severity of the clinical outcome of infection (Jiménez, 2012). Guinea pigs developed severe acute disease when infected with strain NMI (MLVA group IV). Mild to moderate acute infection was induced with strains G and S (MLVA group II) and no acute diseases with strain Dugway (MLVA group III). In CB-17 mice, *C. burnetii* loads in the spleen increased faster after infection with NMI compared to other strains (Russell-Lodrigue et al., 2009), pointing to a direct correlation between replication efficiency of the strains and their virulence. The genetic factors responsible for the genogroup-specific replication await to be identified but several *in vivo* studies have reported strain-specific levels of virulence in rodent models (Hackstadt, 1990; Kazar et al., 1993; Russell-Lodrigue et al., 2009). Even though our findings are in line with these studies, the isolates included in this study displayed some phenotypic variability within genomic groups as, e.g., deduced from induction of pro-inflammatory cytokines in MDM and this was more closely correlated with the invasion rate than with replication efficiency. Replication efficiency of *C. burnetii* strains in turn did not correlate with invasion rate which became particularly apparent for the rodent strain 4 (“Dugway”) showing a high invasion rate but only a moderate replication rate in both MDM models. Therefore, typing methods like MLVA are probably inappropriate to assess the risk potential of isolates or outbreak strains.

Macrophages are the first barrier of the innate immune system to protect the organism from pathogens. During the early phase of infection, i.e., within the first hours, *C. burnetii* induces a rapid expression of pro-inflammatory cytokines, such as IL-1, IL-12, and TNF-α, in bovine (Sobotta et al., 2016) and murine macrophages (Tujulin et al., 1999). In the current study, bovine and human MDM up-regulated expression of pro-inflammatory cytokines IL-1β, IL-12, and TNF-α during the first 3 h *p. i*. in response to infection with different *C. burnetii* strains. Amounts of induced mRNA differed between the strains and seemed to be MLVA genogroup dependent. In bovine and human MDM, group IV isolates caused a strong immune response with a high level of cytokine and chemokine mRNA transcription. In contrast, *C. burnetii* of MLVA group I induced only a weak cellular response. Genotype-specific expression of cytokines was also observed in immunocompetent mice, which were inoculated with different *C. burnetii* strains (Russell-Lodrigue et al., 2009). The inflammatory response observed in that study resembled human acute Q fever infections with an upregulation of TNF and IL-6, but not IL-1β (Capo et al., 1999). In boMDM, active IL-1β protein was also not detected 1 d after stimulation with NM variants (Sobotta et al., 2016) or different *C. burnetii* strains (this study, data not shown). In this regard, responses in the boMDM model are similar to those in the rodent infection model and in human patients (Capo et al., 1999; Russell-Lodrigue et al., 2009). In a recent study by Ammerdorffer et al. it is shown that *C. burnetii* isolates from cattle induce a more pronounced pro-inflammatory cytokine response in human PBMC compared to isolates from infected small ruminants and chronically infected patients (Ammerdorffer et al., 2017). Our results prove only a slight increase of pro-inflammatory cytokine expression in human MDM after infection with bovine *C. burnetii* isolates compared to infection with isolates originating from sheep and goats. Isolates from human, rodent and tick induced in boMDM and huMDM a clearly higher cytokine expression than strains from ruminants. Similar to the data from Ammerdorffer et al. strain 2 (“Henzerling”) isolated from acute human infection also induced a higher expression of cytokines and chemokines in both MDM models than *C. burnetii* strain 3 (“Scurry”) isolated from a chronically infected patient.

Despite *C. burnetii* NMI replication in boMDM in the subsequent days of culture, expression of pro-inflammatory cytokines decreases rapidly to background values (Sobotta et al., 2016). This effect could also be seen with a selection of *C. burnetii* strains (this study, data not shown). Activation of host cells by *C. burnetii* therefore appears to mainly depend on early interactions of the bacteria with the host cell like the attachment process. TLR2 receptors are implicated in the pro-inflammatory response to *C. burnetii* (Meghari et al., 2005). Bacterial membrane proteins, e.g., OmpA (Jeannin et al., 2002) or heat shock proteins (Hsp) (Asea, 2008), interact with TLR2. Both proteins are located on the surface of *C. burnetii* (Macellaro et al., 1998; Martinez et al., 2014), implying that different levels of expression of immunologically active membrane proteins could be a reason for differences between *C. burnetii* strains.

Following bacterial infection, macrophages polarize into M1 or M2 macrophages. M1 macrophages respond to infection with high expression of pro-inflammatory cytokines and surface markers whereas M2 macrophages are poorly microbicidal and provoke an anti-inflammatory immune response (Mosser and Edwards, 2008). Similar to the cytokine response by macrophages, the expression of surface markers is under control of TLR2 or 4 and a result of the contact between the host and bacterial antigens (Biswas et al., 2008). Bacterial membrane proteins such as OmpA or Hsp might not only be involved in increased expression of pro-inflammatory cytokines during the early phase of infection but also in the activation of MDM. *C. burnetii* induced an atypical M2-polarization in murine (Fernandes et al., 2016), bovine (Sobotta et al., 2016) and human macrophages (Benoit et al., 2008a) with a prominent downregulation of typical M1-markers. In the current study, no major changes in the expression of different surface markers after *C. burnetii* infection could be observed. Only human isolate 2 (strain “Henzerling”) induced a pronounced up-regulation of CD40 or CD80 expression. There was no correlation of surface marker expression with genetic properties or origin of bacterial strains. During acute *C. burnetii* infection in humans, an atypical M2 polarization promotes bacterial long-time survival in their hosts (Benoit et al., 2008b). However, limited cell activation in terms of altered cell surface antigen expression in conjunction with strain-specific but only short-lasting up-regulation of pro-inflammatory cytokines exhibited by all *C. burnetii* strains tested in this study strongly argues in favor of a general capability of *C. burnetii* to restrict the immune response in different hosts.

A comparison of open reading frames of selected *C. burnetii* strains unveiled differences but respective genes were either hypothetical or nonfunctional (Beare et al., 2006). During complete genome comparison of NMI, K, G, and Dugway a collection of pseudogenes was found that contribute to pathology-specific virulence of *C. burnetii* (Beare et al., 2009). Combining these genetic details, *in vivo* data from rodent models (Russell-Lodrigue et al., 2009) and our results may reveal the determinants for *C. burnetii* virulence and identify targets for vaccine or therapeutic intervention.

## Author contributions

All authors agree with the content of this work. The work was edited by the authors as follows. KS: designed and interpreted all infection experiments and prepared the manuscript. KH: established the isolation of human MDMs and provided the cells for the experiments. PJ: characterized *Coxiella burnetii* genotypically and revised the manuscript. KK: established the isolation of bovine MDMs and revised the manuscript. CH: provided the *Coxiella burnetii* strains, carried out the TCID_50_ determination and revised the manuscript. CM: created the scientific hypothesis, drafted the outline of the study, interpreted the results, read and edited the manuscript.

### Conflict of interest statement

The reviewer KM declared a shared affiliation, with no collaboration, with the authors KS, KH, and CM to the handling Editor. The other authors declare that the research was conducted in the absence of any commercial or financial relationships that could be construed as a potential conflict of interest.

## References

[B1] Ammerdorffer A. Kuley R. Dinkla A. Joosten L. A. B. Toman R. Roest H. J.. (2017). *Coxiella burnetii* isolates originating from infected cattle induce a more pronounced proinflammatory cytokine response compared to isolates from infected goats and sheep. Pathog. Dis. 75:ftx040. 10.1093/femspd/ftx040 28387835

[B2] Asea A. (2008). Heat shock proteins and toll-like receptors. Handb. Exp. Pharmacol. 183, 111–127. 10.1007/978-3-540-72167-3_6 18071657

[B3] Beare P. A. (2012). Genetic manipulation of *Coxiella burnetii*. Adv. Exp. Med. Biol. 984, 249–271. 10.1007/978-94-007-4315-1_13 22711636

[B4] Beare P. A. Samuel J. E. Howe D. Virtaneva K. Porcella S. F. Heinzen R. A. (2006). Genetic diversity of the Q fever agent, *Coxiella burnetii*, assessed by microarray-based whole-genome comparisons. J. Bacteriol. 188, 2309–2324. 10.1128/JB.188.7.2309-2324.2006 16547017 PMC1428397

[B5] Beare P. A. Unsworth N. Andoh M. Voth D. E. Omsland A. Gilk S. D.. (2009). Comparative genomics reveal extensive transposon-mediated genomic plasticity and diversity among potential effector proteins within the genus *Coxiella*. Infect. Immun. 77, 642–656. 10.1128/IAI.01141-08 19047403 PMC2632050

[B6] Benoit M. Barbarat B. Bernard A. Olive D. Mege J. L. (2008a). *Coxiella Burnetii*, the agent of Q fever, stimulates an atypical M2 activation program in human macrophages. Eur. J. Immunol. 38, 1065–1070. 10.1002/eji.200738067 18350541

[B7] Benoit M. Desnues B. Mege J. L. (2008b). Macrophage polarization in bacterial infections. J. Immunol. 181, 3733–3739. 10.4049/jimmunol.181.6.3733 18768823

[B8] Biswas S. K. Sica A. Lewis C. E. (2008). Plasticity of macrophage function during tumor progression: regulation by distinct molecular mechanisms. J. Immunol. 180, 2011–2017. 10.4049/jimmunol.180.4.2011 18250403

[B9] Capo C. Amirayan N. Ghigo E. Raoult D. Mege J. (1999). Circulating cytokine balance and activation markers of leucocytes in Q fever. Clin. Exp. Immunol. 115, 120–123. 10.1046/j.1365-2249.1999.00786.x 9933430 PMC1905180

[B10] Carcopino X. Raoult D. Bretelle F. Boubli L. Stein A. (2009). Q fever during pregnancy: a cause of poor fetal and maternal outcome. Ann. N. Y. Acad. Sci. 1166, 79–89. 10.1111/j.1749-6632.2009.04519.x 19538266

[B11] Confer A. W. Ayalew S. (2013). The OmpA family of proteins: roles in bacterial pathogenesis and immunity. Vet. Microbiol. 163, 207–222. 10.1016/j.vetmic.2012.08.019 22986056

[B12] Fernandes T. D. Cunha L. D. Ribeiro J. M. Massis L. M. Lima-Junior D. S. Newton H. J.. (2016). Murine Alveolar macrophages are highly susceptible to replication of *Coxiella burnetii* phase II *in vitro*. Infect. Immun. 84, 2439–2448. 10.1128/IAI.00411-16 27297388 PMC4995897

[B13] Frangoulidis D. Rodolakis A. Heiser V. Landt O. Splettstoesser W. Meyer H. (2009). DNA microarray-chip based diagnosis of Q-fever (*Coxiella burnetii*). Clin. Microbiol. Infect. 15(Suppl 2), 165–166. 10.1111/j.1469-0691.2008.02210.x 19281457

[B14] Graham J. G. Macdonald L. J. Hussain S. K. Sharma U. M. Kurten R. C. Voth D. E. (2013). Virulent *Coxiella burnetii* pathotypes productively infect primary human Alveolar macrophages. Cell. Microbiol. 15, 1012–1025. 10.1111/cmi.12096 23279051 PMC3655087

[B15] Hackstadt T. (1990). The role of lipopolysaccharides in the virulence of *Coxiella burnetii*. Ann. N. Y. Acad. Sci. 590, 27–32. 10.1111/j.1749-6632.1990.tb42203.x 2378455

[B16] Hendrix L. R. Samuel J. E. Mallavia L. P. (1991). Differentiation Of *Coxiella burnetii* isolates by analysis of restriction-endonuclease-digested DNA separated by SDS-PAGE. J. Gen. Microbiol. 137, 269–276. 10.1099/00221287-137-2-269 1673152

[B17] Jäger C. Lautenschläger S. Willems H. Baljer G. (2002). *Coxiella burnetii* plasmid types QpDG And QpH1 are closely related and likely identical. Vet. Microbiol. 89, 161–166. 10.1016/S0378-1135(02)00155-4 12243893

[B18] Jäger C. Willems H. Thiele D. Baljer G. (1998). Molecular characterization of *Coxiella burnetii* isolates. Epidemiol. Infect. 120, 157–164. 10.1017/S0950268897008510 9593485 PMC2809385

[B19] Jeannin P. Magistrelli G. Goetsch L. Haeuw J. F. Thieblemont N. Bonnefoy J. Y.. (2002). Outer membrane protein A (OmpA): a new pathogen-associated molecular pattern that interacts with antigen presenting cells-impact on vaccine strategies. Vaccine 20(Suppl 4), A23–A27. 10.1016/S0264-410X(02)00383-3 12477424

[B20] Jiménez P. H. (2012). Genetische Unterschiede Zwischen Coxiella Burnetii-Isolaten Und Ihre Korrelation Zur Epidemiologie Und Klinik Des Q-Fiebers. Giessen: Vvb Laufersweiler Verlag.

[B21] Kazár J. Lesý M. Propper P. Valková D. Brezina R. (1993). Comparison of virulence for guinea pigs and mice of different *Coxiella burnetii* phase I strains. Acta Virol. 37, 437–448. 8010182

[B22] Kersh G. J. Oliver L. D. Self J. S. Fitzpatrick K. A. Massung R. F. (2011). Virulence of pathogenic *Coxiella burnetii* strains after growth in the absence of host cells. Vector Borne Zoonotic Dis. 11, 1433–1438. 10.1089/vbz.2011.0670 21867419

[B23] Macellaro A. Tujulin E. Hjalmarsson K. Norlander L. (1998). Identification of a 71-Kilodalton surface-associated Hsp70 homologue in *Coxiella burnetii*. Infect. Immun. 66, 5882–5888. 9826369 10.1128/iai.66.12.5882-5888.1998 PMC108745

[B24] Mallavia L. P. (1991). Genetics of *Rickettsiae*. Eur. J. Epidemiol. 7, 213–221. 10.1007/BF00145669 1679397

[B25] Martinez E. Cantet F. Fava L. Norville I. Bonazzi M. (2014). Identification of OmpA, a *Coxiella burnetii* protein involved in host cell invasion, by multi-phenotypic high-content screening. PLoS Pathog. 10:e1004013. 10.1371/journal.ppat.1004013 24651569 PMC3961360

[B26] Maurin M. Raoult D. (1999). Q fever. Clin. Microbiol. Rev. 12, 518–553. 10515901 10.1128/cmr.12.4.518 PMC88923

[B27] Meghari S. Honstettre A. Lepidi H. Ryffel B. Raoult D. Mege J. L. (2005). TLR2 is necessary to inflammatory response in *Coxiella burnetii* infection. Ann. N. Y. Acad. Sci. 1063, 161–166. 10.1196/annals.1355.025 16481508

[B28] Mosser D. M. Edwards J. P. (2008). Exploring the full spectrum of macrophage activation. Nat. Rev. Immunol. 8, 958–969. 10.1038/nri2448 19029990 PMC2724991

[B29] Pfaffl M. W. Horgan G. W. Dempfle L. (2002). Relative expression software tool (REST) for group-wise comparison and statistical analysis of relative expression results in real-time PCR. Nucleic Acids Res. 30:e36. 10.1093/nar/30.9.e36 11972351 PMC113859

[B30] Pinet F. Dupont A. Bencherif N. Guihot A. L. Quatannens B. Amouyel P. (2003). Morphology, homogeneity and functionality of human monocytes-derived macrophages. Cell. Mol. Biol. (Noisy-le-grand). 49, 899–905. 14656047

[B31] Russell-Lodrigue K. E. Andoh M. Poels M. W. Shive H. R. Weeks B. R. Zhang G. Q.. (2009). *Coxiella burnetii* isolates cause genogroup-specific virulence in mouse and guinea pig models of acute Q fever. Infect. Immun. 77, 5640–5650. 10.1128/IAI.00851-09 19786560 PMC2786457

[B32] Seshadri R. Paulsen I. T. Eisen J. A. Read T. D. Nelson K. E. Nelson W. C.. (2003). Complete genome sequence of the Q-fever pathogen *Coxiella burnetii*. Proc. Natl. Acad. Sci. U.S.A. 100, 5455–5460. 10.1073/pnas.0931379100 12704232 PMC154366

[B33] Sobotta K. Hillarius K. Mager M. Kerner K. Heydel C. Menge C. (2016). *Coxiella burnetii* infects primary bovine macrophages and limits their host cell response. Infect. Immun. 84, 1722–1734. 10.1128/IAI.01208-15 27021246 PMC4907144

[B34] Soulas C. Baussant T. Aubry J. P. Delneste Y. Barillat N. Caron G.. (2000). Outer membrane protein A (OmpA) binds to and activates human macrophages. J. Immunol. 165, 2335–2340. 10.4049/jimmunol.165.5.2335 10946255

[B35] Svraka S. Toman R. Skultety L. Slaba K. Homan W. L. (2006). Establishment of a genotyping scheme for *Coxiella burnetii*. FEMS Microbiol. Lett. 254, 268–274. 10.1111/j.1574-6968.2005.00036.x 16445755

[B36] Tilburg J. J. Roest H. J. Buffet S. Nabuurs-Franssen M. H. Horrevorts A. M. Raoult D.. (2012). Epidemic genotype of *Coxiella burnetii* among goats, sheep, and humans in the Netherlands. Emerging Infect. Dis. 18, 887–889. 10.3201/eid1805.111907 22516554 PMC3358082

[B37] Tujulin E. Lilliehöök B. Macellaro A. Sjöstedt A. Norlander L. (1999). Early cytokine induction in mouse P388D1 macrophages infected by *Coxiella Burnetii*. Vet. Immunol. Immunopathol. 68, 159–168. 10.1016/S0165-2427(99)00023-9 10438316

[B38] Valková D. Kazár J. (1995). A new plasmid (QpDV) common to *Coxiella burnetii* isolates associated with acute and chronic Q fever. FEMS Microbiol. Lett. 125, 275–280. 10.1111/j.1574-6968.1995.tb07368.x 7875575

[B39] van der Hoek W. Morroy G. Renders N. H. Wever P. C. Hermans M. H. Leenders A. C.. (2012). epidemic Q fever in humans in the Netherlands. Adv. Exp. Med. Biol. 984, 329–364. 10.1007/978-94-007-4315-1_17 22711640

[B40] Voth D. E. Beare P. A. Howe D. Sharma U. M. Samoilis G. Cockrell D. C.. (2011). The *Coxiella burnetii* cryptic plasmid is enriched in genes encoding type iv secretion system substrates. J. Bacteriol. 193, 1493–1503. 10.1128/JB.01359-10 21216993 PMC3067651

[B41] Willems H. Ritter M. Jäger C. Thiele D. (1997). plasmid-homologous sequences in the chromosome of plasmidless *Coxiella burnetii* scurry Q217. J. Bacteriol. 179, 3293–3297. 10.1128/jb.179.10.3293-3297.1997 9150226 PMC179109

[B42] Zhang G. To H. Russell K. E. Hendrix L. R. Yamaguchi T. Fukushi H.. (2005). identification and characterization of an immunodominant 28-kilodalton *Coxiella burnetii* outer membrane protein specific to isolates associated with acute disease. Infect. Immun. 73, 1561–1567. 10.1128/IAI.73.3.1561-1567.2005 15731054 PMC1064944

